# Application of the multidimensional fatigue inventory (MFI-20) in cancer patients receiving radiotherapy.

**DOI:** 10.1038/bjc.1996.42

**Published:** 1996-01

**Authors:** E. M. Smets, B. Garssen, A. Cull, J. C. de Haes

**Affiliations:** University of Amsterdam, Department of Medical Psychology J4, The Netherlands.

## Abstract

In this paper the psychometric properties of the multidimensional fatigue inventory (MFI-20) are established further in cancer patients. The MFI is a 20-item self-report instrument designed to measure fatigue. It covers the following dimensions: general fatigue, physical fatigue, reduced activity, reduced motivation and mental fatigue. The instrument was used in a Dutch and Scottish sample of cancer patients receiving radiotherapy. The dimensional structure was assessed using confirmatory factor analyses (Lisrel's unweighted least-squares method). The hypothesised five-factor model appeared to fit the data in both samples (adjusted goodness of fit; AGFI: 0.97 and 0.98). Internal consistency of the separate scales was good in both the Dutch and Scottish samples with Cronbach's alpha coefficients ranging from 0.79 to 0.93. Construct validity was assessed by correlating the MFI-20 to activities of daily living, anxiety and depression. Significant relations were assumed. Convergent validity was investigated by correlating the MFI scales with a visual analogue scale measuring fatigue and with a fatigue-scale derived from the Rotterdam Symptom Checklist. Results support the validity of the MFI-20. The highly similar results in the Dutch and Scottish sample suggest that the portrayal of fatigue using the MFI-20 is quite robust.


British Journal of Cancer (1996) 73, 241-245
© 1996 Stockton Press All rights reserved 0007-0920/96 $12.00

### Application of the multidimensional fatigue inventory (MFI-20) in cancer patients receiving radiotherapy

EMA Smets[1], B Garssen[2], A Cull[3] and JCJM de Haes[1,4]

[1]*Academic Medical Centre, University of Amsterdam, Department of Medical Psychology J4, PO Box 22700, 1100 DE Amsterdam, The Netherlands;* [2]*The Helen Dowling Institute for Biopsychosocial Medicine, Rotterdam, The Netherlands;* [3]*ICRF Medical Oncology Unit, Western General Hospital, Edinburgh, UK;* [4]*Department of Clinical Oncology/Medical Decision Making Unit, University of Leiden, The Netherlands.*

**Summary** In this paper the psychometric properties of the multidimensional fatigue inventory (MFI-20) are established further in cancer patients. The MFI is a 20-item self-report instrument designed to measure fatigue. It covers the following dimensions: general fatigue, physical fatigue, reduced activity, reduced motivation and mental fatigue. The instrument was used in a Dutch and Scottish sample of cancer patients receiving radiotherapy. The dimensional structure was assessed using confirmatory factor analyses (Lisrel's unweighted least-squares method). The hypothesised five-factor model appeared to fit the data in both samples (adjusted goodness of fit; AGFI: 0.97 and 0.98). Internal consistency of the separate scales was good in both the Dutch and Scottish samples with Cronbach's alpha coefficients ranging from 0.79 to 0.93. Construct validity was assessed by correlating the MFI-20 to activities of daily living, anxiety and depression. Significant relations were assumed. Convergent validity was investigated by correlating the MFI scales with a visual analogue scale measuring fatigue and with a fatigue-scale derived from the Rotterdam Symptom Checklist. Results support the validity of the MFI-20. The highly similar results in the Dutch and Scottish sample suggest that the portrayal of fatigure using the MFI-20 is quite robust.

**Keywords:** fatigue; assessment; validity; radiotherapy

Fatigue is probably the most frequently reported symptom of patients with cancer. It can be experienced before diagnosis, signalling to the individual the possibility of disease. Following cancer diagnosis, treatments such as surgery, radiotherapy or chemotherapy may induce fatigue. It is estimated that about 70% of cancer patients experience fatigue during radiotherapy and chemotherapy (Smets *et al.*, 1993). In general, fatigue decreases during the period of convalescence. However, results have been reported indicating that some patients continue to experience a lack of energy long after treatment has ended (Fobair *et al.*, 1986; Devlen *et al.*, 1987; Berglund *et al.*, 1991).

The possible consequences of fatigue are reflected in its detrimental effect on activity level. In female chemotherapy patients fatigue and weakness were the symptoms that interfered most with self-care activities (Rhodes *et al.*, 1988). To limit the expenditure of energy, activities and work were scheduled, non-essential activities were decreased and patients reported an increasing dependence on others for home management activities, including meal preparation, grocery shopping and cleaning. In a similar sample, Jamar (1989) found social activities to be decreased as a result of fatigue. Some data suggest that loss of energy or physical stamina affects the ability to work after treatment for cancer (Fobair *et al.*, 1986; Weis *et al.*, 1992). Finally, Bloom and colleagues (1990) found that in male patients treated for Hodgkin's disease perceived energy level interacted with leisure activities but not with work activity.

Studies investigating pyschological distress of cancer patients suggest a relationship between fatigue and negative affect. Fatigue was found to be significantly related to negative mood (Jamar, 1989; Blesch *et al.*, 1991) and to emotional distress experienced during chemotherapy treatment (Nerenz *et al.*, 1982). Patients whose energy level had not returned to normal after treatment for Hodgkin's disease were also more likely to have elevated depression scores (Fobair *et al.*, 1986).

Despite its high prevalence and potential negative effect on patients' activities and emotional well-being, research in fatigue is still underdeveloped. Reliable and valid indicators of fatigue are necessary to study this symptom.

Based on a review of instruments used in studies involving cancer patients it was concluded that most measures of fatigue in cancer are incorporated in instruments that measure broader aspects of patient functioning (Smets *et al.*, 1993). Instruments that are more comprehensive and easy to administer and that have been thoroughly tested for their psychometric properties were found to be lacking. Therefore, we developed a self-report instrument. Taking the position that fatigue is a multidimensional concept, the questionnaire was designed to cover the following dimensions: general expressions of fatigue, physical fatigue, reduced activity, reduced motivation and mental fatigue. These dimensions were based on the manner in which fatigue can be expressed as indicated in the literature and resulting from pilot in-depth patient interviews. The use of a multidimensional instrument offered the opportunity to obtain a profile of fatigue that would give the most adequate description of the experience of the respondents and that might discriminate between populations, moments in time or factors contributing to the fatigue experience. The procedure followed to develop the questionnaire – The Multidimensional Fatigue Inventory (MFI) – and the studies investigating its psychometric properties in different populations have been reported in detail elsewhere (Smets *et al.*, 1995). Here, we give a brief overview of these studies and their results.

The initial version was tested for its structure, internal consistency and validity in a heterogeneous group of patients receiving radiotherapy for cancer ($n$=111). The instrument was tested in patients with chronic fatigue syndrome (CFS) ($n$=357), in psychology students ($n$=481) and medical students ($n$=158), in army recruits ($n$=316) and in junior physicians ($n$=46). The factor structure of the MFI was investigated using confirmatory factor analyses. Results confirmed the proposed five dimensions of fatigue and a similar five-factor solution was supported across all samples tested. The internal consistency of the resulting five scales was good with an average Cronbach's alpha coefficient of 0.80 (range: 0.53–0.93). Construct validity of the instrument was determined by comparing groups with assumed differences in

Correspondence: EMA Smets
Received 9 February 1995; revised 19 June 1995; accepted 23 August 1995

fatigue. For example, patients were expected to be more fatigued than students; soldiers were expected to be more fatigued during a physically intensive military training programme than during their stay in the barrack and junior physicians were expected to report more fatigue during training in internal medicine than before this training period. The scales for general and physical fatigue showed consistent and distinct differences between and within groups according to expectations. General fatigue especially, appeared to be sensitive to predicted changes in fatigue level. The CFS patients and cancer patients differed from the comparison groups as expected on most scales. The scale for reduced motivation behaved as expected in most tests. Finally, mental fatigue seemed to differ somewhat from the other scales, especially in the group of radiotherapy patients. Radiotherapy patients scored lowest on mental fatigue as compared with any of the other groups, which was contrary to expectations (mean score = 10.06, s.d. = 6.1). Convergent validity was assessed by correlating the MFI scales with the scores on a visual analogue scale covering the intensity of fatigue in the sample of radiotherapy patients. Correlations were all significant, ranging from 0.77 for general fatigue ($P<0.001$) to 0.23 for mental fatigue ($P<0.01$). Overall, the psychometric properties of the instrument were promising.

Since our original purpose was to develop a fatigue questionnaire to be used in cancer patient populations, the MFI was tested in two additional investigations involving Dutch and Scottish cancer patients treated with radiotherapy. In this paper results are presented regarding the psychometric properties of the MFI when used in cancer patients treated with radiotherapy: its structure and internal consistency were again assessed and additional information on its validity was obtained. The application of the instrument in both a Dutch and Scottish sample offered the opportunity of a cross-cultural comparison.

### Method

### *Subjects and data collection procedure*

The Dutch sample involved patients in their last week of radiation treatment ($n=141$). Patients were given a letter inviting them to participate, a copy of the questionnaire and a return envelope. They were asked to fill in the questionnaire within the next 3 days. The questionnaire was returned by 98 of these patients (70%).

The Scottish sample involved 134 cancer patients that were treated with radiotherapy at the Western General Hospital in Edinburgh. They were invited to fill in the questionnaire either at the department in the waiting area, or at home. In the latter case, they were instructed to complete the forms on their own, within 24 h. Eighteen patients (13%) failed to return the questionnaire, resulting in a final sample of 116 patients.

### *Instruments*

The MFI-20 consists of five scales, based on different modes of expressing fatigue. 'General fatigue' includes general statements concerning a person's functioning such as 'I feel rested'. 'Physical fatigue' refers to the physical sensation related to the feeling of tiredness. Possible somatic symptoms of fatigue such as light-headedness or sore muscles are not included in this scale in order to exclude as much as possible contamination with the symptoms of somatic illness, independent of fatigue. Reduction in activities and lack of motivation to start any activity are covered by the scales 'reduced activities' and 'reduced motivation' respectively. Finally, cognitive symptoms such as having difficulties concentrating are included in the scale for 'mental fatigue'.

Each scale contains four items for which the person had to indicate on a seven-point scale to what extent the particular statement applies to him or her. An equal number of items is worded in a positive and in a negative direction to counteract response tendencies. A sample from each of the five scales is presented in Figure 1.

The MFI-20 was translated into English by a Dutch professional translator and a native English speaker with a thorough knowledge of Dutch. Both versions were compared and revised by the English investigator. This version was then sent back to the first author, and a final version was made in collaboration with the professional translator.

Besides the MFI, the questionnaire in both studies included the Rotterdam Symptom Checklist (RSCL). This instrument is developed to assess the physical and psychological symptoms experienced by cancer patients, as well as the patient's activities of daily living and overall quality of life (de Haes *et al.*, 1990).

The Scottish patients also completed the Hospital Anxiety and Depression Scale (HADS). The HADS is a self-report questionnaire developed to assess anxiety and depression for use in non-psychiatric hospital settings (Zigmond and Snaith, 1983). A major advantage of this questionnaire over many other depression scales is that it has no items referring directly to feelings of tiredness or sleepiness, which most likely would contaminate the relation with a fatigue

| | | | | | | | | |
|---|---|---|---|---|---|---|---|---|
| 1. I feel fit | Yes, that is true | ☐ | ☐ | ☐ | ☐ | ☐ | No, that is not true |
| 2. Physically I feel only able to do a little | Yes, that is true | ☐ | ☐ | ☐ | ☐ | ☐ | No, that is not true |
| 3. I feel very active | Yes, that is true | ☐ | ☐ | ☐ | ☐ | ☐ | No, that is not true |
| 4. I am not up to much | Yes, that is true | ☐ | ☐ | ☐ | ☐ | ☐ | No, that is not true |
| 5. Thinking requires effort | Yes, that is true | ☐ | ☐ | ☐ | ☐ | ☐ | No, that is not true |

[1] Investigators interested in using the instrument should contact the first author. For academical use, permission will be granted at no charge, but while still under development, investigators will be requested to share their results with the instrument so that reliability and validity testing can proceed appropriately.
[2] Based on the response distribution obtained in the studies presented in this paper and in prior investigations, the response format in the latest version of the MFI-20 has been changed from a seven to a five-point scale.

**Figure 1** Examples of items included in the MFI.

questionnaire. The only item which may refer to fatigue is the item 'I feel as if I am slowed down'. Patients had to indicate their fatigue on a visual analogue scale (VAS) covering the overall intensity of fatigue experienced during the previous days, by placing a mark on a 100 mm line with two end points: 'not at all tired' and 'extremely tired'.

### *Statistical methods*

Confirmatory factor analyses were performed to investigate the structure of the MFI-20. An advantage of confirmatory factor analysis over exploratory factor analysis is that the investigator may specify beforehand the kind of interpretation preferred on theoretical grounds. To assess to which degree and probability the hypothesised model fitted the data, Lisrell's VII unweighted least-squares method was used (Jörekog and Sörbom, 1988). As in the previous studies, it was assumed that fatigue can best be described with five dimensions. Indices of the fit of the model include the chi-square statistic, the goodness of fit index (GFI) and the adjusted goodness of fit index (AGFI). A model that fits the data will have a low chi-square statistic and high GFI and AGFI. The latter two can range from 0 to 1, with indexes of 0.90 or higher indicating a good fit.

Cronbach's alpha coefficients were calculated for the separate scales, for both samples. The alpha coefficient provides an indication of internal consistency, that is the degree of convergence between different items hypothesised to represent the same construct.

Convergent validity refers to the extent to which different ways of measuring the same trait intercorrelate with one another. Convergent validity of the MFI-20 was determined in the Scottish sample by correlating the scales with the VAS. Since the VAS measures a global sensation of fatigue it was expected that the highest correlations would be obtained with the MFI-scale for general fatigue.

Construct validity is evaluated by testing hypotheses about how the instrument should behave. Fatigue is frequently associated with a reduction in activity level. Therefore, an indication of the validity of the instrument was provided by its correlation with the RSCL scale measuring activities of daily living (ADL). A moderate but significant correlation was expected, in particular for the MFI-scale of reduced activity.

Fatigue is also generally acknowledged to be related to negative emotions, in particular depression. Therefore, correlation coefficients were calculated with the HADS scales, assuming a high correlation with both scales, but more so for depression than for anxiety. It was also hypothesised that the association with depression would be strongest for the MFI scale of reduced motivation. In order to investigate whether an association beween fatigue and depression would primarily result from the overlap in item content, analyses involving the depression scale were performed twice: once with the total score and once with the exclusion of the item 'I feel as if I am slowed down'.

To investigate whether the MFI-20 is capable of distinguishing between groups with different fatigue intensities, two groups were formed based on the scores on the RSCL item 'tiredness'; those who report no or little tiredness, and those who report moderate or very much tiredness. Differences in mean MFI scores were tested using univariate analyses of variance.

### Results

### *Characteristics of the patients*

The Dutch sample included 52 women (54%) and 45 men (46%). Their average age was 61 years (range: 20–88). The most frequent diagnoses were cancer of the lungs (28%) and breast (19%). The majority of patients in the Scottish sample was female (88 women = 77% and 28 men = 23%). The average age was 57 years (range: 14–85). The two most prevalent diagnoses in this sample were breast cancer (46%) and cancer of the urogenital tract (13%).

### *Intensity of fatigue*

An indication of the intensity of fatigue in these samples is obtained by the frequency distribution of responses to the items tiredness and lack of energy in the RSCL. In the Dutch sample 54% of the patients reported moderate or severe tiredness and 31% lack of energy. In the Scottish sample these percentages were 40% and 35% respectively. In both samples these percentages were higher than for any of the other somatic symptoms included in the questionnaire.

### *Psychometric properties of the MFI-20*

The confirmatory factor analyses resulted in an AGFI of 0.98 (chi-square 528.11, d.f. = 160) in the Dutch sample and 0.97 (chi-square 364.84, d.f. = 160) in the Scottish sample. Both these AGFIs are well above 0.90, which indicates a good fit of the five-factor model on the data. These findings confirm the appropriateness of distinguishing five dimensions of fatigue in the MFI. The intercorrelations of these scales are presented in Table I.

Regarding the internal consistency, the Cronbach's alphas (Table II) are good (>0.75) to excellent (>0.90) for these five scales in both samples.

Convergent validity of the MFI-20 in the Scottish sample was substantiated, as correlations between the separate scales and the VAS are all significant ($P<0.001$), as expected. The correlations were 0.83 for general fatigue, 0.71 for physical fatigue, 0.67 for reduced activity, 0.61 for reduced motivation and 0.54 for mental fatigue. As hypothesised, the scale for general fatigue had the highest correlation with the VAS.

Construct validity was assessed next, for each of the MFI scales. Results regarding the correlations with activities of daily living and emotional distress are presented in Table III, for both samples. The significant correlations between the first four MFI scales and the ADL scale confirm the expectations. It was also assumed that the strongest relation would be obtained with reduced activity. This assumption is only partly supported. In both samples, the relationships between the first four fatigue dimensions and the scale for activities of daily living are similar. Only mental fatigue has substantially lower correlation coefficients.

As expected, the associations between anxiety and depression and fatigue are significant. The relation with

**Table I** Intercorrelations between the separate MFI scales for the Dutch and Scottish samples

| | *Dutch sample* n = *90* | | | | | *Scottish sample* n = *107* | | | | |
|---|---|---|---|---|---|---|---|---|---|---|
| | *1* | *2* | *3* | *4* | *5* | *1* | *2* | *3* | *4* | *5* |
| 1. General fatigue | – | | | | | – | | | | |
| 2. Physical fatigue | 0.86** | – | | | | 0.84** | – | | | |
| 3. Reduced activity | 0.78** | 0.87** | – | | | 0.79** | 0.73** | – | | |
| 4. Reduced motivation | 0.72** | 0.76** | 0.77** | – | | 0.71** | 0.66** | 0.76** | – | |
| 5. Mental fatigue | 0.38** | 0.30* | 0.39** | 0.36** | – | 0.58** | 0.53** | 0.55** | 0.53** | – |

*P < 0.01, **P < 0.001.

Table II Descriptive information for the separate MFI scales in the Dutch and Scottish samples: mean scores, standard deviations, percentage with maximum and minimum scores and Cronbach's alphas

| | Dutch sample | | | | | Scottish sample | | | | |
|---|---|---|---|---|---|---|---|---|---|---|
| | *Mean* | *s.d.* | *Percentage with maximum score* | *Percentage with minimum score* | *Cronbach's alpha* | *Mean* | *s.d.* | *Percentage with maximum score* | *Percentage with minimum score* | *Cronbach's alpha* |
| General fatigue | 19.9 | 8.5 | 28.6 | 9.2 | 0.93 | 15.8 | 8.2 | 9.7 | 11.2 | 0.85 |
| Physical fatigue | 18.2 | 8.8 | 16.3 | 11.2 | 0.90 | 14.2 | 7.6 | 3.7 | 14.2 | 0.79 |
| Reduced activity | 19.4 | 8.5 | 23.5 | 10.2 | 0.89 | 17.2 | 8.5 | 15.7 | 10.4 | 0.87 |
| Reduced motivation | 17.3 | 8.4 | 15.3 | 12.2 | 0.83 | 11.9 | 7.3 | 4.5 | 19.4 | 0.79 |
| Mental fatigue | 14.2 | 8.0 | 10.2 | 17.3 | 0.82 | 11.1 | 8.2 | 5.2 | 33.6 | 0.89 |

Table III Correlations between MFI scales and activities of daily living (RSCL); anxiety and depression (HADS)

| | *Dutch sample* | *Scottish sample* | | | | | |
|---|---|---|---|---|---|---|---|
| | *ADL* n=*61* | *ADL* n=*76* | *Anxiety* n=*108* | *95% confidence interval*[a] | *Depression* n=*109* | *95% confidence interval*[a] | *Depression minus item 8* |
| General fatigue | 0.58** | 0.42** | 0.51** | 0.36 < 0.64 | 0.77** | 0.67 < 0.83 | 0.67** |
| Physical fatigue | 0.71** | 0.44** | 0.45** | 0.28 < 0.59 | 0.67** | 0.70 < 0.85 | 0.56** |
| Reduced activity | 0.67** | 0.43** | 0.40** | 0.23 < 0.55 | 0.70** | 0.59 < 0.78 | 0.62** |
| Reduced motivation | 0.59** | 0.37* | 0.41** | 0.25 < 0.56 | 0.71** | 0.61 < 0.79 | 0.67** |
| Mental fatigue | 0.28 | 0.19 | 0.52** | 0.37 < 0.65 | 0.61** | 0.48 < 0.72 | 0.58** |

[a]Confidence intervals are calculated using Fischer's $Z$-transformation procedure. *$P < 0.01$, **$P < 0.001$.

Table IV Mean scores for the MFI scales for 'tired' and 'not tired' patients as indicated by their scores on the RSCL item 'tiredness'

| | *Dutch sample* (n = 94) | | | | *Scottish sample* (n = 109) | | | |
|---|---|---|---|---|---|---|---|---|
| | *Not tired* | | *Tired* | | *Not tired* | | *Tired* | |
| | *Mean* | *s.d.* | *Mean* | *s.d.* | *Mean* | *s.d.* | *Mean* | *s.d.* |
| General fatigue | 12.68 | 7.09 | 25.45* | 4.17 | 10.38 | 6.84 | 21.36* | 5.14 |
| Physical fatigue | 16.22 | 11.57 | 34.10* | 7.83 | 10.04 | 6.48 | 18.31* | 6.33 |
| Reduced activity | 15.51 | 8.97 | 29.32* | 6.55 | 12.30 | 7.80 | 21.96* | 6.07 |
| Reduced motivation | 11.79 | 7.17 | 21.80* | 6.37 | 8.43 | 5.10 | 15.71* | 7.48 |
| Mental fatigue | 14.48 | 9.13 | 19.51 | 9.88 | 7.83 | 5.76 | 14.42* | 8.92 |

*The difference in mean score between 'not tired' and 'tired' patients is significant ($P < 0.001$).

depression is stronger than the relation with anxiety. The confidence intervals demonstrate that the differences in correlations between anxiety and depression are most likely significant, except for mental fatigue. This finding also supports our hypothesis. The assumption that the association with depression would be strongest for reduced motivation is not supported by the data. The relationship is of similar strength for all five scales. The exclusion of the item 'I feel as if I am slowed down' from the HADS depression scale results in a decrease of the correlations for all five scales. This change in correlation coefficients is greater than with the removal of any of the other items of the depression scale.

The average scores of the MFI scales for 'tired' *vs* 'not tired' patients in both samples, are presented in Table IV.

The differences in MFI scale scores between these two groups are all significant with the exception of the scores on mental fatigue in the Dutch sample.

### Discussion

The results favour the psychometric properties of the MFI-20. Its hypothesised structure of five dimensions is supported in both samples. The internal consistencies of the resulting scales are all highly satisfactory.

Both convergent and construct validity of the MFI-20 were assessed in these studies. Convergent validity of the English version of the MFI-20 was supported by the significant correlations with the VAS in the Scottish sample. A similar result was obtained in a previous study involving Dutch radiotherapy patients in which the MFI was correlated with a VAS (Smets *et al.*, 1995).

Construct validity is satisfactory across both samples as indicated by the significant correlations with the daily activity scores, depression and anxiety. The direction of these relations is as anticipated: a higher fatigue score is associated with a reduction in activity and with increased emotional distress.

The findings in the Dutch and Scottish samples regarding structure, internal consistency and validity of the instrument are very similar. This suggests that cancer patients from different cultural and linguistic backgrounds respond to rating their fatigue in a similar fashion. However, the result show differences between the two samples in average scale scores. First, these differences might reflect cultural differences in item content or item endorsement, and do not reflect a true difference in fatigue experience. Another explanation is based on the difference in the distribution of diagnoses between the two samples. Almost half of the Scottish sample consists of women with breast cancer, whereas a quarter of the Dutch sample consists of patients with carcinoma of the lungs. It is our experience that lung cancer patients are generally more fatigued than breast cancer patients. Two final explanations come with the difference in data accrual procedure between both samples. Dutch patients were all approached during the last week of their treatment when fatigue was assumed to be at its peak. The Scottish sample included patients at different moments of the treatment process, presumably representing a greater variety of fatigue intensities, which could be reflected in lower average scores. The floor and ceiling scores for the two samples as reported in Table II show that the percentage of patients with the maximum score of 28 is higher in the Dutch sample, as expected. However, the standard deviations for the two samples are similar. This indicates that the overall distribution of scores in the two groups is similar. It appears that the Dutch sample includes a subgroup with extreme maximum scores.

Another difference in procedure concerned the fact that the Dutch patients were asked to complete the questionnaire at home, within 3 days, whereas the majority of the Scottish patients completed the questionnaire at the radiotherapy department, immediately after being invited to participate. This procedural difference may explain the lower response rate in the Dutch sample (70% *vs* 87%) and may have resulted in selective drop-out of the least fatigued patients. Unfortunately, for ethical reasons, no information was available on the non-responders.

Two additional findings from the studies presented require discussion. First, with the exception of mental fatigue, all MFI scales behave more or less similarly. In general, the correlations between the separate MFI scales and with other measures do not differ significantly from each other. Consequently, although the MFI-20 consists of separate dimensions, as indicated by the confirmatory factor analyses, four of these dimensions appear to be equally important for the fatigue experience of cancer patients receiving radiotherapy. The similarity in their relation with other constructs, and the high inter-scale correlations suggest that maybe the distinction between at least some of these dimensions is not so relevant. Similar results have been found in previous investigations. For the moment, however, we have decided to retain the five scales until more information is available on the behaviour of the separate scales in other patient populations and in relation to other constructs. If it turns out that using separate scales does not provide additional information, scales may be combined in a revised version of the questionnaire.

The second point in need of discussion is the magnitude of the correlations of the MFI scales with depression. Even with the removal of an item from the depression scale because of its reference to fatigue the correlations remain substantial. This raises the issue of whether the MFI-20 is capable of discriminating between fatigue and depression. The relation between fatigue and depression is notoriously complicated and has attracted a lot of attention in the literature in particular with respect to the chronic fatigue syndrome. In cancer, feelings of depression may result from the fact that one has a possibly fatal disease, and a depressed state of mind may induce fatigue. However, depression could not only be a cause, but also a result of persistent feelings of tiredness. Loss of function and loss of energy resulting directly from the disease or its treatment may have its adverse consequences. Finally, depression and fatigue may co-occur in cancer because both result from the same biological factors (Hayes, 1991). Additional research is required to assess discriminant validity of the MFI scales with regard to depression in cancer patients. A design would be needed with repeated measurements in a situation in which one would expect fatigue and depression to change in different directions or at least to a different degree. Unfortunately, such situations are scarce. However, if the results of fatigue and depression diverge, this would support the validity of the fatigue instrument, although we would always expect to find a significant association between fatigue and depression. As indicated, future research with the MFI should address its discriminant validity with respect to depression. It should also address the instrument's responsiveness to change. Sensitivity is an important attribute of a symptom questionnaire when it is to be used in clinical trials. The currently used cross-sectional design does not provide any information on the sensitivity of the scales. Investigating responsiveness to change would require a longitudinal design in which change in fatigue levels is expected as a result of treatment or disease progression. Our previous study in healthy subjects demonstrated that the scale for general fatigue is most sensitive to changes in fatigue level.

Future use of the instrument in other cancer patient populations, other patient groups or non-patient populations has to result in normative data that help to interpret scale scores and scale score differences.

So far, the results regarding the structure, the inter-scale relations and internal consistencies of the MFI scales are in line with the results obtained in the psychometric studies performed previously (Smets *et al.*, 1995). Again the scale for mental fatigue behaves somewhat differently than the other scales. The resemblance in results indicates that the portrayal of fatigue using the MFI-20 is quite robust. Its use for descriptive purposes will hopefully contribute to a better conceptual understanding of this complex experience in cancer patients. In clinical practise the possibility of obtaining a profile of fatigue might help to identify subgroups of patients who are particularly at risk for certain 'types' of fatigue, and it may enhance the ability to plan and evaluate therapy. When, for example in clinical trials, a short instrument is required, it could be argued that only the scale for general fatigue is used.

**Acknowledgements**
This work was supported by the Dutch Cancer Society, grant: IKA 92-137. We are grateful to the Departments of Radiotherapy of the Academic Hospital Leiden and the Western General Hospital in Edinburgh for their contribution to this investigation.

### References

BERGLUND G, BOLUND C, FORNANDER T, RUTQVIST LE AND SJÖDEN P-O. (1991). Late effects of adjuvant chemotherapy and postoperative radiotherapy on quality of life among breast cancer patients. *Eur. J. Cancer*, **27** 1075–1081.

BLESCH K, PAICE JA, WICKHAM R, HARTE N, SCHNOOR DK, PURL S, REHWAIT M, KOPP P, MANSON S, COVENEY S, MCHALE M AND CAHILL M. (1991). Correlates of fatigue in people with breast or lung cancer. *Oncol. Nurs. Forum*, **18**, 81–87.

BLOOM JR, GORSKY RD, FOBAIR P, HOPPE R, COX RS, VARGHESE A AND SPIEGEL D. (1990). Physical performance at work and at leisure: Validation of a measure of biological energy in survivors of Hodgkin's disease. *J. Psychosoc. Oncol.*, **8**, 49–63.

DEVLEN J, MAGUIRE P, PHILIPS P, CROWTHER D AND CHAMBERS H. (1987). Psychological problems associated with diagnosis and treatment of lymphomas. 1. retrospective; 2. prospective. *Br. Med. J.*, **295**, 953–957.

FOBAIR P, HOPPE RT, BLOOM J, COX R, VAUGHESE A AND SPIEGEL D. (1986). Psychosocial problems among survivors of Hodgkin's disease. *J. Clin. Oncol.*, **4**, 805–814.

HAES JCJM DE, KNIPPENBERG FCE VAN AND NEIJT JP. (1990). Measuring psychological and physical distress in cancer patients: structure and application of the Rotterdam Symptom Checklist. *Br. J. Cancer*, **62**, 1034–38.

HAYES JR. (1991). Depression and chronic fatigue in cancer patients. *Primary Care*, **18**, 327–339.

JAMAR S. (1989). Fatigue in women receiving chemotherapy for ovarian cancer. In Funk SG, Tonquist EM, Campagne MT, Archer Gopp L and Wiese RA (eds). *Key Aspects of Comfort. Management of Pain Fatigue and Nausea*. pp. 224–228. Springer: New York.

JÖRESKOG KG AND SÖRBOM D. (1988). *LISREL VII; A Guide to the Program and Applications*. Spss: Chicago.

NERENZ DR, LEVENTHAL H AND LOVE RR. (1982). Factors contributing to emotional distress during cancer chemotherapy. *Cancer*, **50**, 1020–1027.

RHODES VA, WATSON PM AND HANSON BM. (1988). Patients' descriptions of the influence of tiredness and weakness on self-care abilities. *Cancer Nursing*, **11**, 186–94.

SMETS EMA, GARSSEN B, SCHUSTER-UITTERHOEVE ALJ AND DE HAES JCJM. (1993). Fatigue in cancer patients. *Br. J. Cancer*, **68**, 220–224.

SMETS EMA, GARSSEN B, BONKE B AND DE HAES JCJM. (1995). The Multidimensional Fatigue Inventory (MFI); Psychometric qualities of an instrument to assess fatigue. *J. Psychosom. Res.*, **39**, 315–325.

WEIS J, KOCH U AND GELDSETZER M. (1992). Changes in vocation after cancer: Empirical study in vocational rehabilitation (1992). *Soz Präventivmed.*, **37**, 85–95.

ZIGMOND AS AND SNAITH RP. (1983). The hospital anxiety and depression scale. *Acta Psychiatr. Scand.*, **67**, 361–370.


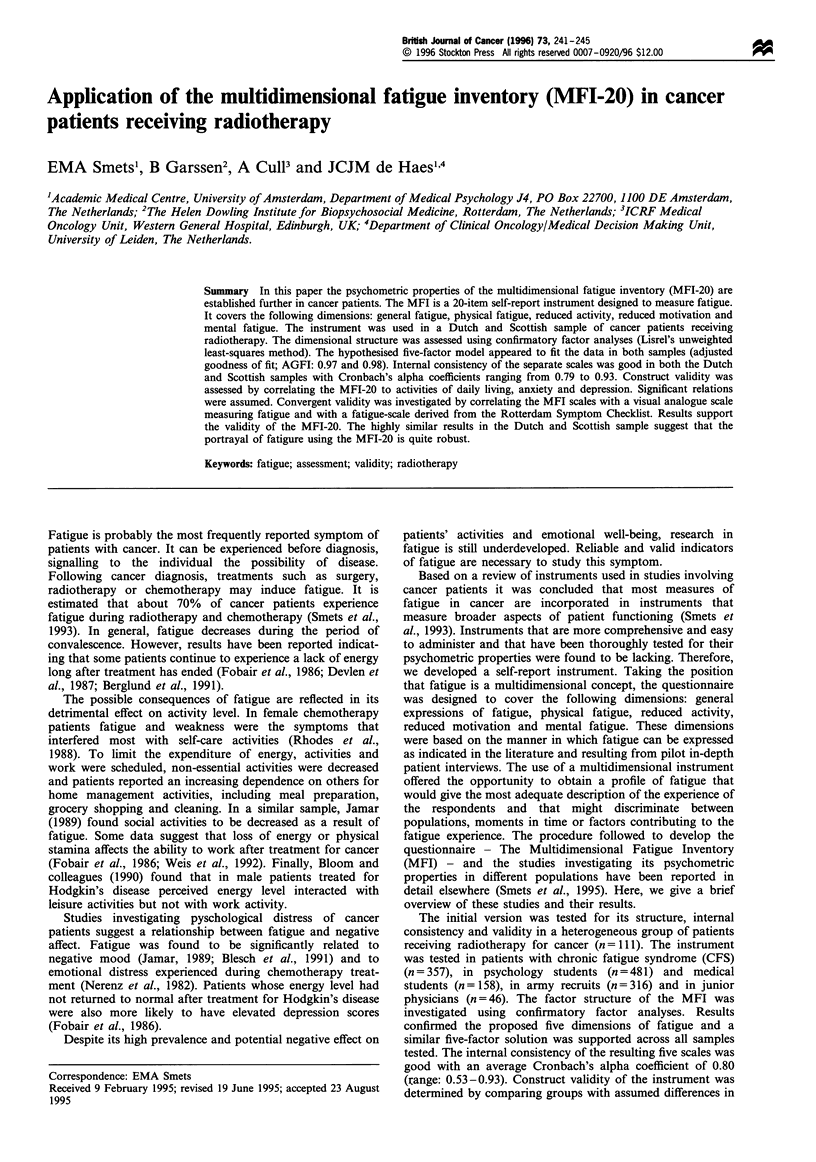

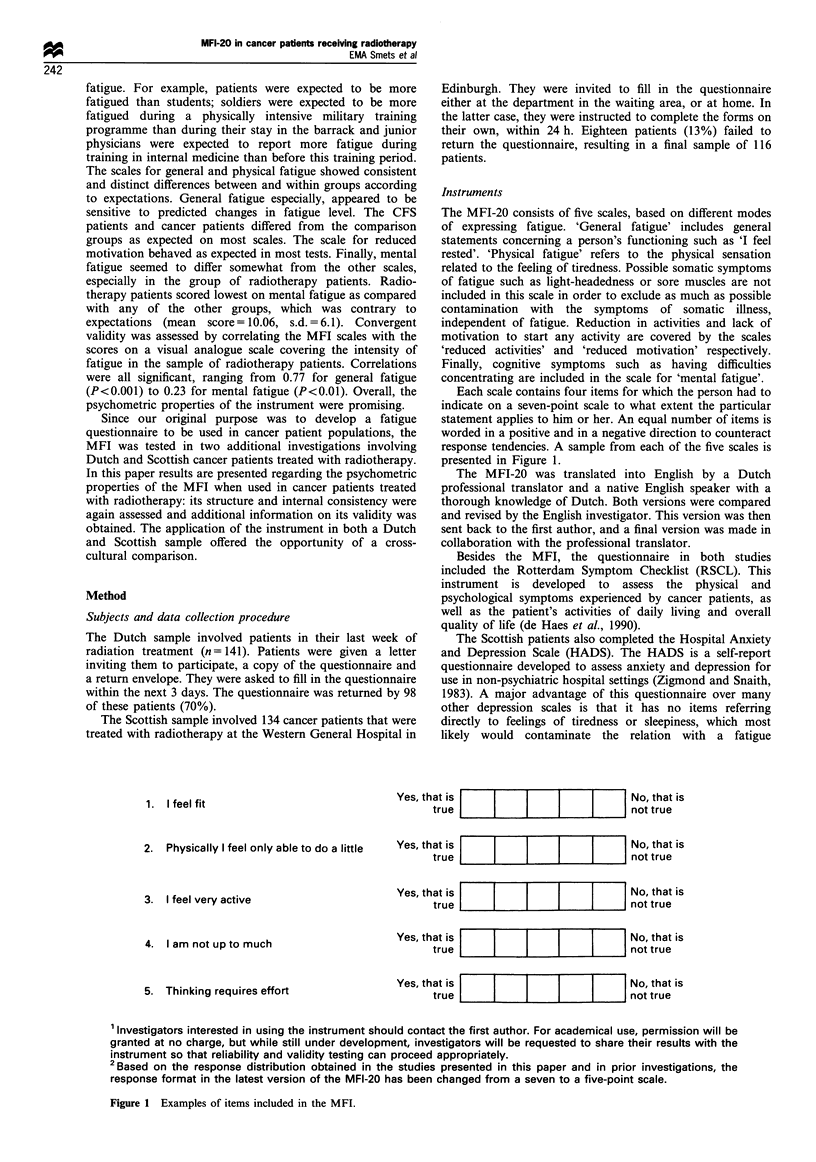

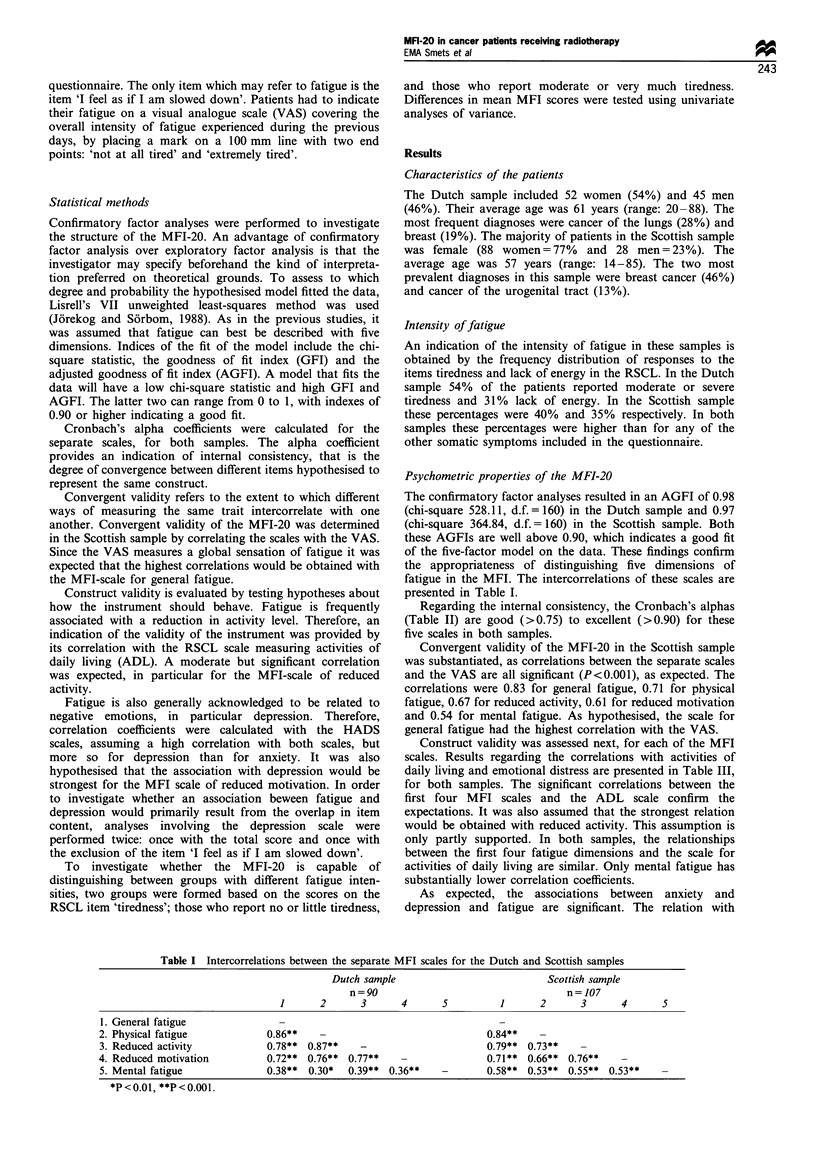

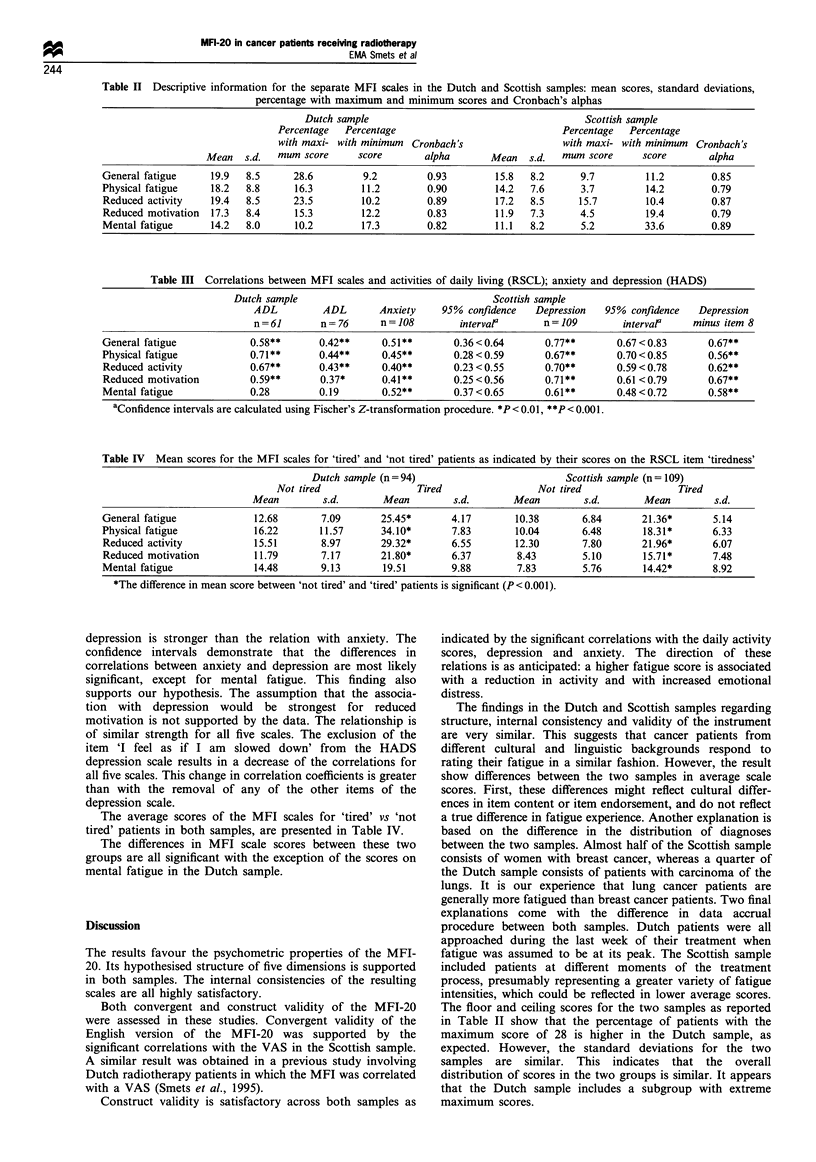

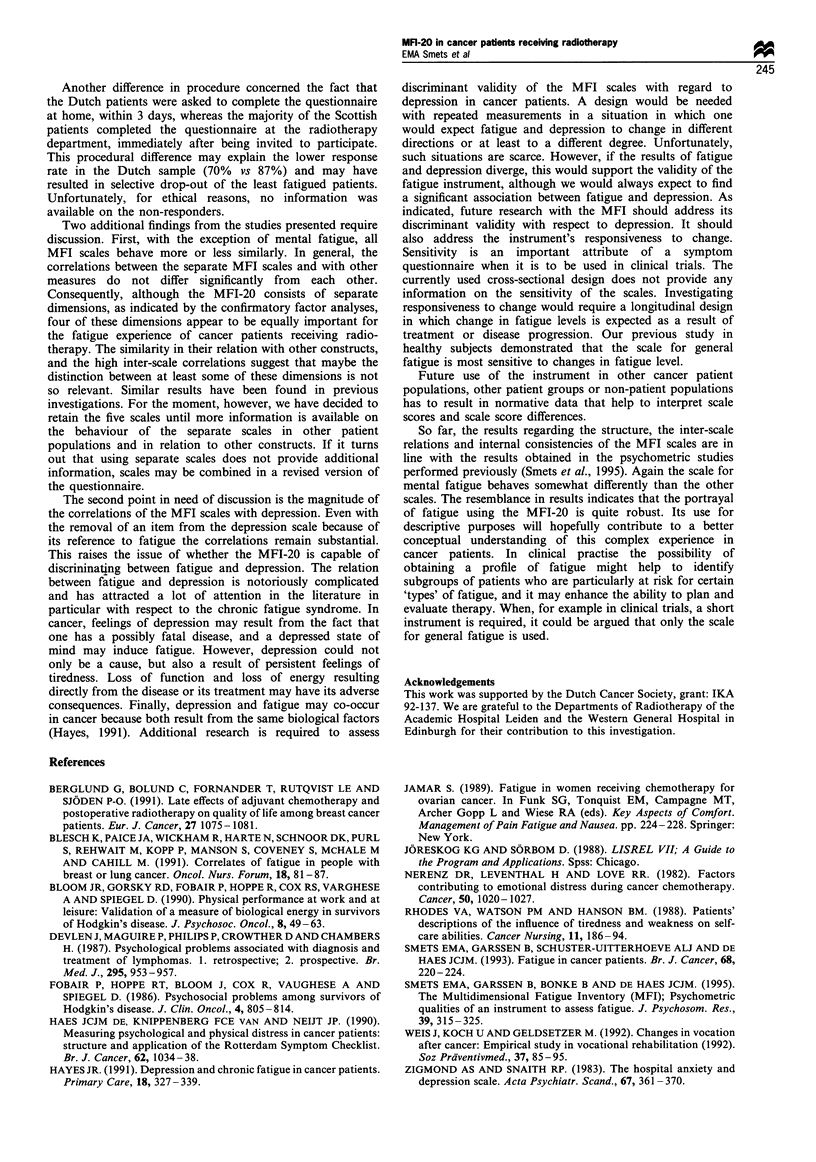


## References

[OCR_00679] Berglund G., Bolund C., Fornander T., Rutqvist L. E., Sjödén P. O. (1991). Late effects of adjuvant chemotherapy and postoperative radiotherapy on quality of life among breast cancer patients.. Eur J Cancer.

[OCR_00685] Blesch K. S., Paice J. A., Wickham R., Harte N., Schnoor D. K., Purl S., Rehwalt M., Kopp P. L., Manson S., Coveny S. B. (1991). Correlates of fatigue in people with breast or lung cancer.. Oncol Nurs Forum.

[OCR_00697] Devlen J., Maguire P., Phillips P., Crowther D., Chambers H. (1987). Psychological problems associated with diagnosis and treatment of lymphomas. I: Retrospective study.. Br Med J (Clin Res Ed).

[OCR_00704] Fobair P., Hoppe R. T., Bloom J., Cox R., Varghese A., Spiegel D. (1986). Psychosocial problems among survivors of Hodgkin's disease.. J Clin Oncol.

[OCR_00714] Hayes J. R. (1991). Depression and chronic fatigue in cancer patients.. Prim Care.

[OCR_00729] Nerenz D. R., Leventhal H., Love R. R. (1982). Factors contributing to emotional distress during cancer chemotherapy.. Cancer.

[OCR_00732] Rhodes V. A., Watson P. M., Hanson B. M. (1988). Patients' descriptions of the influence of tiredness and weakness on self-care abilities.. Cancer Nurs.

[OCR_00742] Smets E. M., Garssen B., Bonke B., De Haes J. C. (1995). The Multidimensional Fatigue Inventory (MFI) psychometric qualities of an instrument to assess fatigue.. J Psychosom Res.

[OCR_00737] Smets E. M., Garssen B., Schuster-Uitterhoeve A. L., de Haes J. C. (1993). Fatigue in cancer patients.. Br J Cancer.

[OCR_00753] Zigmond A. S., Snaith R. P. (1983). The hospital anxiety and depression scale.. Acta Psychiatr Scand.

[OCR_00708] de Haes J. C., van Knippenberg F. C., Neijt J. P. (1990). Measuring psychological and physical distress in cancer patients: structure and application of the Rotterdam Symptom Checklist.. Br J Cancer.

